# Theophylline Intoxication-Like Signs Despite Normal Serum Concentration in an Older Patient

**DOI:** 10.7759/cureus.33017

**Published:** 2022-12-27

**Authors:** Masaatsu Kuwahara

**Affiliations:** 1 Department of Emergency Medicine, Takarazuka City Hospital, Takarazuka, JPN

**Keywords:** theophylline, metabolic acidosis, dyspnea, anti-asthmatic agents, bronchodilator agents

## Abstract

Theophylline is a bronchodilator with a narrow therapeutic index. Theophylline toxicity can manifest as metabolic acidosis, hypokalemia, arrhythmia, and other life-threatening symptoms. A 90-year-old Asian woman with a 10-day history of hyporexia presented to the emergency room with shortness of breath and low SpO_2_. Diagnostic tests revealed ketosis, metabolic acidosis, hypokalemia, and hypercalcemia. Diabetic ketoacidosis and alcoholic ketoacidosis were ruled out based on the patient’s history and laboratory parameters. When it was discovered that the patient had been previously prescribed theophylline, theophylline toxicity was suspected, despite a serum concentration of 16.6 μg/mL, which was within the range typically considered safe. She received symptomatic infusion therapy and corrective treatment for electrolyte abnormalities and was discharged 15 days later. Theophylline intoxication can occur even when serum concentrations do not exceed the therapeutic range, and the severity may be higher among older patients.

## Introduction

Theophylline is a bronchodilator used to treat airway diseases. Its use has declined in the United States in favor of more effective agents, such as inhaled corticosteroids and β2-agonists; however, it is commonly used for bronchial asthma in Japan and other countries [1]. Theophylline has a narrow therapeutic index owing to the narrow window between the effective serum concentrations and concentrations associated with toxicity, which may manifest as metabolic acidosis, hypokalemia, arrhythmias, and other life-threatening clinical effects. Therapeutic serum levels of theophylline are typically reported as 10-20 μg/mL, but here we describe a rare case of an older adult presenting with clinical signs highly indicative of severe theophylline intoxication despite serum concentrations within the therapeutic range.

## Case presentation

The patient was a 90-year-old Asian woman residing in a nursing home with a 10-day history of hyporexia. She was brought to the emergency room for dyspnea, and her SpO_2_ on room air was 80% on arrival. After oxygen supplementation (10 L/minute), her SpO_2_ increased to 99%; however, the patient was hypotensive (blood pressure 89/36 mmHg), tachycardic (145 beats per minute), and tachypneic (30 breaths per minute). Her Glasgow Coma Scale score was 11 (E4V1M6); she was in shock and showed signs of impaired consciousness. Her skin turgor was diminished, and symptomatic infusion therapy was initiated after a point-of-care ECHO revealed the collapse of the inferior vena cava.

Arterial blood gas analysis at the presentation revealed the following: pH, 7.135; arterial partial pressure of carbon dioxide, 12.6 mmHg; bicarbonate, 4.1 mmol/L; base excess, −24.2 mmol/L; anion gap, 28.5 mmol/L; lactic acid, 25.0 mg/dL; and high-anion gap metabolic acidosis. Urinary ketone levels were 4+, suggesting ketosis. The blood glucose level was moderately high at 166 mg/dL (normal: 73-109 mg/dl), serum potassium levels were decreased at 2.7 mmol/L (normal: 3.6-4.8 mmol/L), and serum ionized calcium ion levels were high at 1.44 mmol/L (normal: 1.15-1.29 mmol/L). Liver function test results were unremarkable: aspartate aminotransferase level, 15 U/L (normal: 13-30 U/L); alanine aminotransferase level, 2 U/L (normal: 10-42 U/L); and prothrombin time and international normalized ratio: 0.93 (Table 1).

**Table 1:** Blood data at time of admission pCO_2_: arterial partial pressure of carbon dioxide, AST: aspartate aminotransferase level, ALT: alanine aminotransferase level, PT-INR: prothrombin time and international normalized ratio

	Blood data at time of admission	Reference range
pH	7.135	7.35–7.45
pCO_2_	12.6 mmHg	35–45mmHg
Bicarbonate	4.1 mmol/L	21–28mmol/L
Base excess	−24.2 mmol/L	−2 to 3mmol/L
Anion gap	28.5 mmol/L	10–20mmol/L
Potassium levels	2.7 mmol/L	3.6–4.8mmol/L
Ionized calcium ion levels	1.44 mmol/L	1.15–1.29mmol/L
Blood glucose level	166 mg/dL	73–109mg/dL
Lactic acid	25 mg/dL	4.5–14.4mg/dL
Urinary ketone levels	4+	
AST	15 U/L	13–30U/L
ALT	2 U/L	10–42U/L
PT-INR	0.93	

Diabetic ketoacidosis was ruled out based on the patient’s blood glucose levels and the absence of oral sodium-glucose co-transporter-2 inhibitors; alcoholic ketoacidosis was also ruled out because the patient had not consumed alcohol. The patient’s decreased appetite was believed to have caused the ketosis. Metabolic acidosis was caused by both lactic acidosis and ketoacidosis.

The patient was then found to have been prescribed an extended-release formulation of theophylline (200 mg/day) for bronchial asthma at another hospital for over five years.

At this point, her clinical signs, particularly shock, metabolic acidosis, hypokalemia, and hypercalcemia, combined with her medical history, led us to consider theophylline toxicity. Notably, however, her serum theophylline concentration was 16.6 μg/mL, which was within the reference range (10-20 μg/mL). The patient received infusion therapy (1500 mL/day) to correct dehydration as well as replacement therapy comprising 330 mEq potassium and 80 mmol phosphorus to restore electrolyte balance. Her general condition improved, and she was discharged on the 15th day of hospitalization.

## Discussion

This case report describes an older patient who presented with clinical signs of theophylline toxicity despite a serum theophylline concentration within the range typically considered to be safe (10-20 μg/mL) [2]. Theophylline poisoning can be acute or chronic. In acute poisoning, serum theophylline concentration correlates with the severity of the resulting clinical signs, but this is not necessarily true for chronic poisoning. In most cases of chronic poisoning, serum theophylline concentrations equaled or exceeded 20 μg/mL. However, this report shows that poisoning is possible even at concentrations of 10-20 μg/mL [3]. The relationship between the intensity and severity of clinical signs of theophylline intoxication and actual serum theophylline concentrations is unclear in cases of chronic poisoning. Older patients are more likely to experience life-threatening events due to chronic theophylline poisoning, and it is impossible to infer the extent of clinical signs based on peak serum theophylline concentrations [4]. Adults older than 75 years are 16.7 times more likely to be severely affected than those younger than 25 years, regardless of serum theophylline concentration [4].

Theophylline activity arises from adenosine receptor antagonism and indirect adrenergic activity. Adenosine receptors are distributed throughout the body, and antagonism of these receptors results in both therapeutic and toxic effects, including bronchodilation, tachycardia, arrhythmias, seizures, and cerebral vasoconstriction. Theophylline intoxication is reportedly associated with increased plasma catecholamines [5-7]. Excess catecholamines may contribute to metabolic abnormalities such as hypokalemia, metabolic acidosis, arrhythmias, and hypotension.

Theophylline is metabolized primarily in the liver by the enzyme cytochrome P450 1A2. Various factors affect theophylline metabolism; for example, clearance may be reduced due to advanced age, hepatic impairment, or interactions with other drugs [8]. No evidence of hepatic impairment was seen with our patient, and there was no history of concomitant use of drugs that either inhibit or enhance theophylline metabolism, thus leaving age as the most likely risk factor. As older adults have a lower reserve capacity, the severity of complications may be significantly amplified [9]. However, the findings of this study are limited because serum theophylline concentrations were only measured once at admission, and theophylline toxicity is rare. Further research on possible associations between age, toxicity threshold, and severity of clinical signs should be conducted.

## Conclusions

The present case suggests that theophylline intoxication should not be simply ruled out based on a serum concentration within the range typically considered safe. Furthermore, advanced age may be a critical risk factor for toxicity and increased severity of complications. Although theophylline is no longer the primary drug for asthma treatment in several countries, it is commonly used worldwide. Therefore, clinicians should be familiar with the signs of theophylline toxicity and aware of its potential idiosyncrasies.
